# Supplementary figures and images for: Smartphone tristimulus colorimetry for skin-tone analysis at common pulse oximetry anatomical sites
Source: Biophotonics Discov. 2025 May 19;2(3):032504. doi: 10.1117/1.BIOS.2.3.032504 (PMC13052481; doi:10.1117/1.BIOS.2.3.032504)

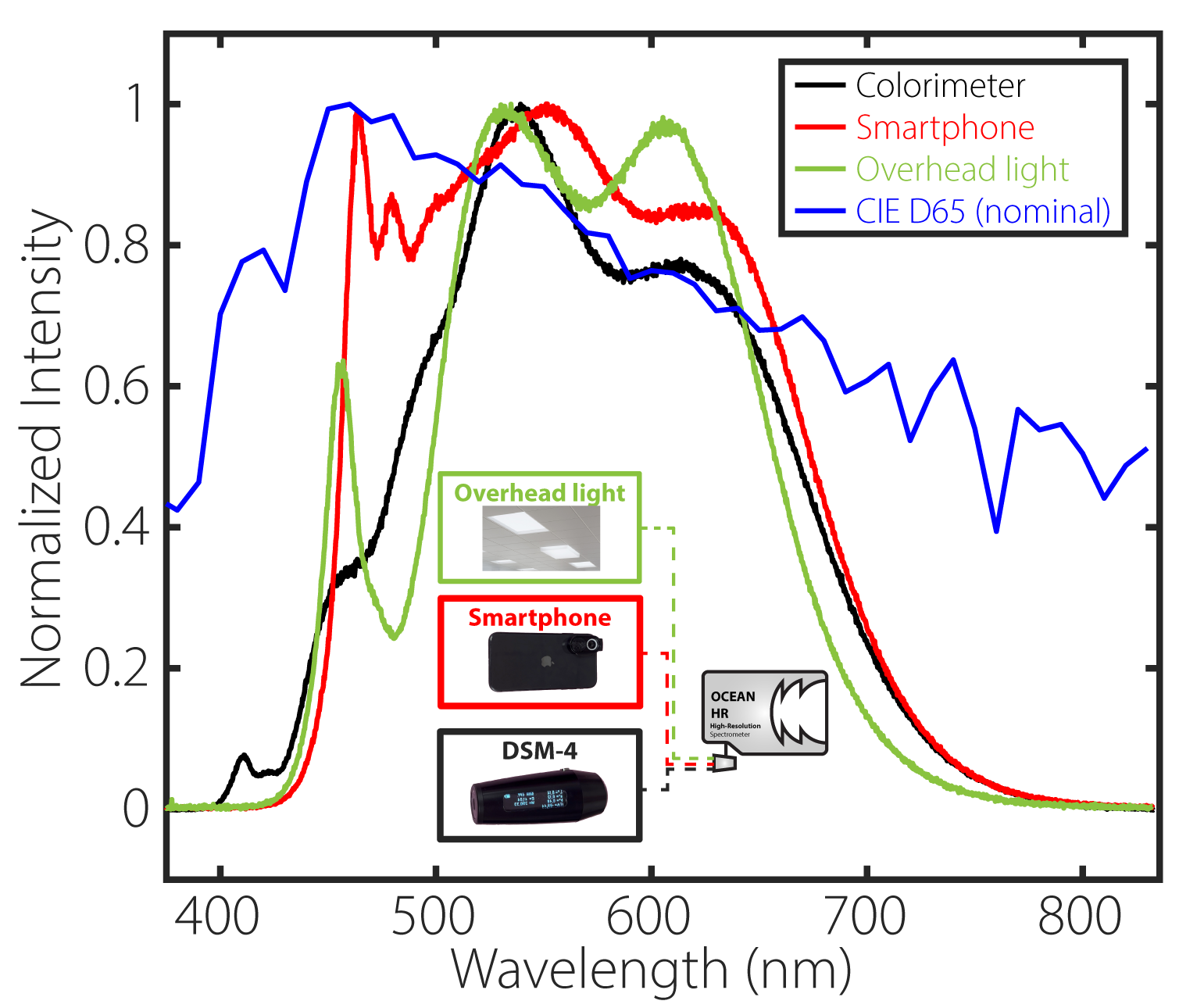

Supplement: Supplementary file 1 [file BIOS-002-032504_SD001.png]

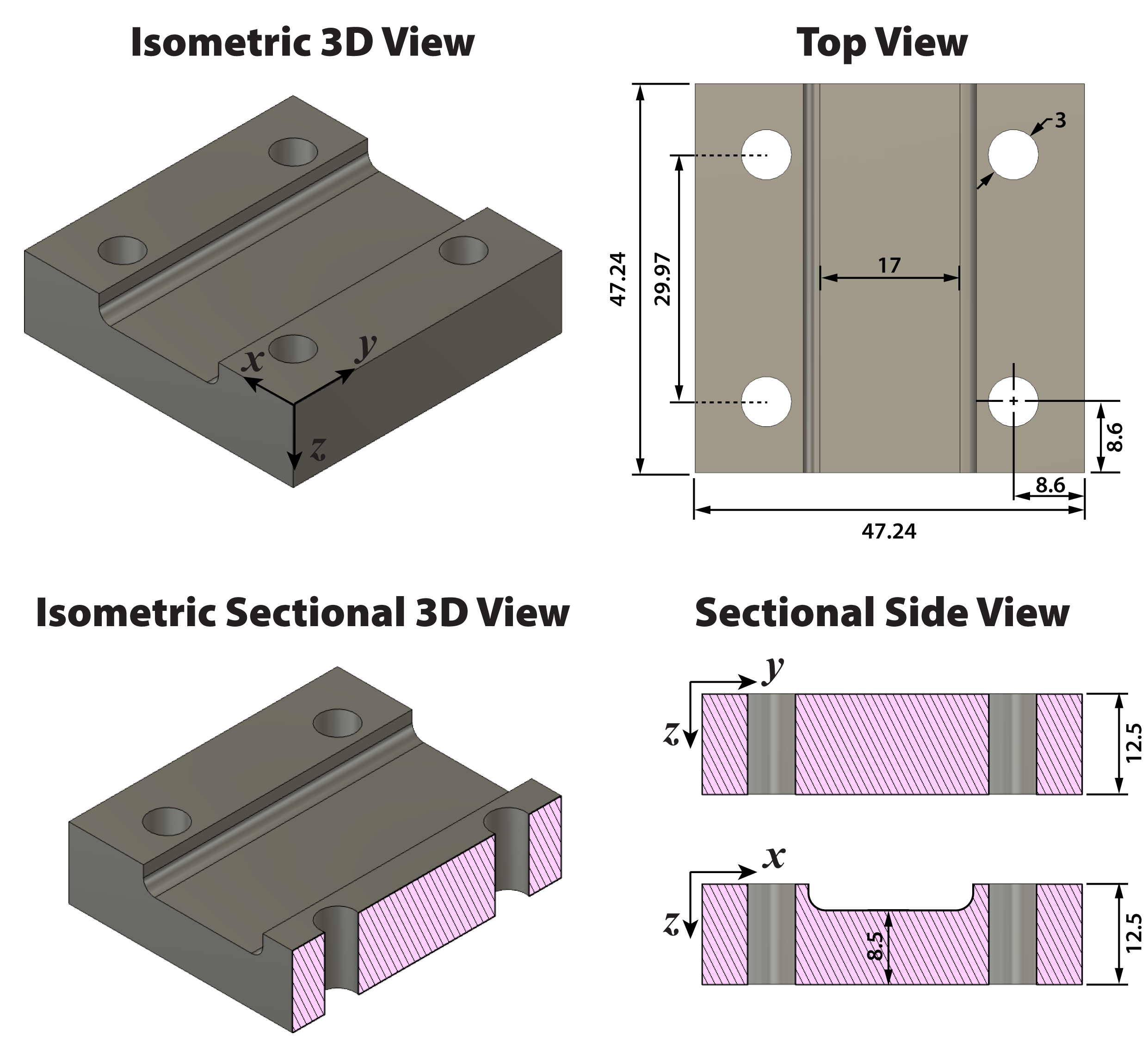

Supplement: Supplementary file 2 [file BIOS-002-032504_SD002.png]

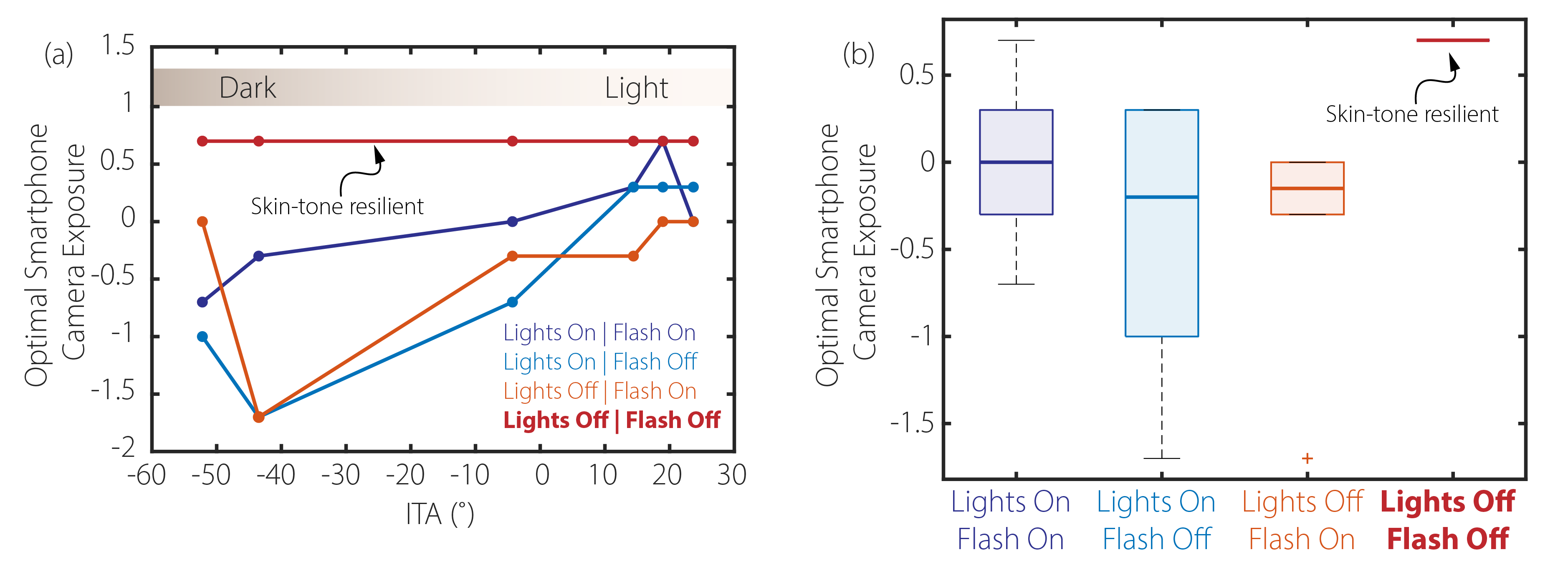

Supplement: Supplementary file 3 [file BIOS-002-032504_SD003.png]
